# Hepatic but not brain iron is rapidly chelated by deferasirox in aceruloplasminemia due to a novel gene mutation

**DOI:** 10.1016/j.jhep.2010.04.039

**Published:** 2010-12

**Authors:** Armin Finkenstedt, Elisabeth Wolf, Elmar Höfner, Bethina Isasi Gasser, Sylvia Bösch, Rania Bakry, Marc Creus, Christian Kremser, Michael Schocke, Milan Theurl, Patrizia Moser, Melanie Schranz, Guenther Bonn, Werner Poewe, Wolfgang Vogel, Andreas R. Janecke, Heinz Zoller

**Affiliations:** 1 Department of Medicine II Gastroenterology and Hepatology, Medical University of Innsbruck, Innsbruck, Austria; 2 Department of Neurology, Medical University of Innsbruck, Innsbruck, Austria; 3 Department of Analytical Chemistry & Radiochemistry, Leopold Franzens University of Innsbruck, Innsbruck, Austria; 4 Department of Chemistry, University of Basel, Basel, Switzerland; 5 Department of Radiology I, Medical University of Innsbruck, Innsbruck, Austria; 6 Department of Ophthalmology, Medical University of Innsbruck, Innsbruck, Austria; 7 Department of Pathology, Medical University of Innsbruck, Innsbruck, Austria; 8 Department of Pediatrics II, Medical University of Innsbruck, Innsbruck, Austria; 9 Department of Human Genetics, Medical University of Innsbruck, Innsbruck, Austria

**Keywords:** ACP, aceruloplasmin, CP, ceruloplasmin, GPI, glycosylphosphatidylinositol, PCR, polymerase chain reaction, RT-PCR, reverse transcription PCR, MRI, magnetic resonance imaging, NAFLD, non alcoholic fatty liver disease, DMS, dysmetabolic siderosis, Bp, basepairs, Metabolic liver disease, Iron chelation, Iron overload, Fibrosis, Neurodegeneration

## Abstract

**Background & Aims:**

Aceruloplasminemia is a rare autosomal recessive neurodegenerative disease associated with brain and liver iron accumulation which typically presents with movement disorders, retinal degeneration, and diabetes mellitus. Ceruloplasmin is a multi-copper ferroxidase that is secreted into plasma and facilitates cellular iron export and iron binding to transferrin.

**Results:**

A novel homozygous ceruloplasmin gene mutation, c.2554+1G>T, was identified as the cause of aceruloplasminemia in three affected siblings. Two siblings presented with movement disorders and diabetes. Complementary DNA sequencing showed that this mutation causes skipping of exon 14 and deletion of amino acids 809–852 while preserving the open reading frame. Western blotting of liver extracts and sera of affected patients showed retention of the abnormal protein in the liver. Aceruloplasminemia was associated with severe brain and liver iron overload, where hepatic mRNA expression of the iron hormone hepcidin was increased, corresponding to the degree of iron overload. Hepatic iron concentration normalized after 3 and 5 months of iron chelation therapy with deferasirox, which was also associated with reduced insulin demands. During short term treatment there was no clinical or imaging evidence for significant effects on brain iron overload.

**Conclusions:**

Aceruloplasminemia can show an incomplete clinical penetrance but is invariably associated with iron accumulation in the liver and in the brain. Iron accumulation in aceruloplasminemia is a result of defective cellular iron export, where hepcidin regulation is appropriate for the degree of iron overload. Iron chelation with deferasirox was effective in mobilizing hepatic iron but has no effect on brain iron.

## Introduction

Aceruloplasminemia (ACP) is a rare autosomal recessive disorder of iron metabolism, where affected patients typically present with a triad of diabetes mellitus, retinal degeneration, and neurological symptoms [1]. Absence of ceruloplasmin (CP) in plasma in combination with mild anemia, hypoferremia, and hyperferritinemia is a diagnostic finding.

The prevalence of ACP is highest in Japan where it is estimated to be 1 in 2 million [2]. ACP can be caused by nonsense, missense, splice-site or frame shift mutations in the CP gene, where a total of about 40 different mutations has been described [3,4]. One ACP patient, heterozygous for only one missense *CP* mutation p.R701W, has been reported suggesting a dominant negative effect of this mutation [5].

CP is mainly expressed in hepatocytes and glial cells, where the hepatic splicing variant includes exons 1–19 and encodes a soluble, 1040 amino acid protein [6]. Astrocytes express a membrane-bound glycosylphosphatidylinositol (GPI) anchored form of CP, resulting from alternative splicing, where a cryptic splice site within exon 18 is spliced to exon 20 and exon 19 is skipped [6]. Hepatic CP is secreted into the plasma as a glycosylated metalloprotein that binds >90% of the copper in plasma. CP is a multi-copper ferroxidase which facilitates iron binding to transferrin and promotes iron export from hepatocytes, macrophages, and glial cells [7–9]. Iron export from macrophages is required for the recycling of heme–iron from senescent red blood cells. In contrast, iron export from hepatocytes supplies iron from stores during iron deprivation or iron loss [10]. The only known iron-exporter is ferroportin, which is stabilized by GPI-anchored CP in glial cells [9]. How soluble CP in plasma promotes iron export from hepatocytes remains unknown. Ferroxidase activity and stabilization of ferroportin at the plasma-membrane require copper in the active site of CP [9]. Supply of copper to the hepatocellular Golgi apparatus for correct CP assembly and folding requires the activities of ATP7A, which is implicated in intestinal copper uptake, as well as the activity of ATP7B, which is an intracellular copper transport protein [11,12]. Menkes disease and Wilson’s disease, which are associated with genetic defects in ATP7A and ATP7B, respectively, are characteristically associated with low serum CP concentrations resulting from reduced copper supply to the Golgi apparatus [4].

Iron accumulation in ACP affects the liver, pancreas, and central nervous system, where the retina, striatum, thalamus, and dentate nucleus are primarily affected. Brain iron accumulation can be associated with retinal degeneration, extrapyramidal symptoms, cerebellar symptoms, and mental dysfunction which indicate neurodegeneration [3]. In contrast, hepatic iron overload is not associated with liver disease in ACP but diabetes may be the result of iron-induced damage of pancreatic islet cells [13]. Disease onset typically occurs in the 4th–5th decade of life and ACP has a slowly progressive course, with deterioration of neurological symptoms that can cause typical complications and death in most patients in the 6th decade of life [3].

Iron chelation therapy with deferoxamine or deferiprone has been reported to reduce hepatic iron stores in ACP and transfusion of fresh plasma has been shown to partially reconstitute plasma ferroxidase activity [14–18]. Brain iron concentrations and neurological symptoms did not improve on iron chelators, which may be a result of the limited permeability of the blood–brain barrier for these drugs [14,15].

Herein, we report the clinical presentation of ACP in three siblings and the biochemical consequences of a novel *CP* mutation in a canonical splice-site. Further, the clinical, biochemical, and radiological responses to iron chelation therapy with deferasirox are presented.

## Material and methods

### Serum iron parameters, copper, ceruloplasmin, and ferroxidase determination

This study was approved by our local ethics committee and informed consent was obtained from all patients.

Serum iron parameters, serum copper concentration, serum CP concentrations, and hematological parameters were determined using routine clinical biochemical methods.

Serum ferroxidase activity was determined using *p*-Phenylenediamine (Sigma Aldrich, Vienna, Austria) as artificial substrate according to a previously published method [19].

### CP gene analysis

For cDNA sequencing, total liver RNA extracted from a liver biopsy specimen of the index case was used as a PCR template after reverse transcription. PCR products were directly sequenced using the BigDye Terminator Kit v.3.2 (Applied Biosystems, Vienna, Austria) and an ABI Prism 3130 Genetic Analyzer (Applied Biosystems, Vienna, Austria). Primers and annealing temperatures for RT-PCR are listed in Supplementary Table 1.

Exons 11–17 and the exon–intron boundaries of the CP gene were sequenced from genomic DNA. Primer sequences are listed in Supplementary Table 2. Sequence analysis was carried out with the Sequencher v4.9 software (Genecodes, Ann Arbor, MI) using the Ensemble entries for human CP as reference (ENSG00000047457, ENST00000264613).

To screen 100 chromosomes from healthy controls for the presence of an eventually identified c.2554+1G>T mutation, a restriction fragment length polymorphism assay was set up. The newly identified c.2554+1G>T mutation creates an additional RsaI cleavage cut site in the corresponding PCR product, which can be used for high throughput screening. PCR products were analyzed after RsaI digestion by HPLC using a WAVE DHPLC system (Transgenomic, Glasgow, UK).

### Liver biopsy processing and MRI studies

Sections of formalin-fixed paraffin-embedded liver tissue were stained with hematoxylin and eosin and with Perls’ reagents according to standard methods. Tissue iron and copper concentrations were determined in an unfixed and air-dried liver biopsy sample by atomic absorption spectroscopy.

For the non-invasive quantification of liver iron, T2∗ relaxation times were calculated from a fat-saturated multi-echo gradient-echo MR sequence. For the quantification of liver T1-values a fast T1-mapping sequence based on an inversion recovery snapshot FLASH sequence was used which allowed the acquisition of a single T1 map during one breath hold [20,21]. T1 and T2∗ acquisitions were obtained for identical slice positions and slice parameters. T2∗ and T1 maps were calculated off-line using ImageJ software (NIH, MD, USA). The parameter maps were analyzed by the placement of region of interests into different hepatic lobe segments, whereby identical regions of interests were used in T1 and T2∗ maps. Care was taken to avoid the inclusion of large vessels.

### Immunoblotting of the liver extracts

Protein extracts from liver biopsies were made by the addition of radio-immuno precipitation buffer-containing protease inhibitors to a 1 mm^3^ snap-frozen piece of liver tissue. After reduction and heat denaturation, protein extracts were separated on an 8% discontinuous SDS gel. After blotting onto a PVDF membrane, CP immunoreactivity was detected by incubation of blocked (in 1% skim milk in PBS containing 0.1% Tween) membranes in goat anti human CP polyclonal antibody (C0911-Sigma Aldrich, Vienna, Austria) diluted 1:100 in blocking buffer. Immune complexes were visualized by ECL after incubation of washed membranes in a rabbit anti goat peroxidase conjugate at a 1:1000 dilution.

### Homology modelling

A 3D homology model of truncated CP protein was made based on the PDB template 2J5WA using the Swiss-Model workspace [22]. Models were analyzed and figures were prepared with Accelrys DS Visualizer v2.0.

## Results

### Case report

The index case (IV.2) was a 47 year old male who complained of a progressive gait disorder with falls which had deteriorated over the last 3–4 years. In the neurological examination the patient presented with a cerebellar syndrome with a slurred speech, hypometric saccades, and an intention tremor in the right hand. When standing he was unsteady with a reduced postural stability and a pronounced gait ataxia. The patient also had a left-sided spastic hemiparesis following a traumatic brain injury at the age of 23 years. Insulin-dependent diabetes mellitus was diagnosed at the age of 26 years.

Blood tests showed marked hyperferritinemia and hypoferremia with normal transferrin but decreased transferrin saturation (Table 1). Serum CP and ferroxidase activity were undetectable. Serum copper was 2.1 μM (reference range 11–22 μM). No hematological or other biochemical abnormalities were found.

### MRI studies and liver histology

To assess tissue iron concentrations, MRI of the abdomen and the brain with T1 and T2∗ relaxation time-mapping was carried out. As shown in Fig. 2A, iron accumulation was found in the liver, the basal ganglia, and the dentate nuclei. Liver histology showed a normally structured liver with severe hepatocellular iron overload but no stainable iron in macrophages (Fig. 3). The results from hepatic iron quantification are shown in Table 1. Based on these findings the clinical diagnosis of ACP was made in the proband and family studies were initiated.

### Family study

**Fig. 1:**
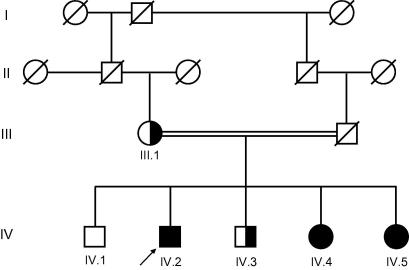
**Pedigree of the affected family**. Three children of a consanguineous marriage are affected by ACP, one sibling and the mother are heterozygous carriers of c.2554+1G>T. The index case is marked by an arrow.

Family studies showed a consanguineous loop and the family history revealed diabetes in a sister (IV.4) of the proband (Fig. 1). As shown in Table 1 hyperferritinemia, hypoferremia, and undetectable serum CP in two sisters indicated ACP. A movement disorder with a hypomimia and a postural tremor of both arms were present in the older sister (IV.4), whereas all other family members were clinically unaffected. Ophthalmological investigation in all individuals showed retinal pigment epithelium changes with a central depigmentation and a bulls-eye like macula but a normal vision and perimetry in patients IV.4 and IV.5. MR imaging and liver histology in the biochemically affected sisters showed iron accumulation in the basal ganglia, dentate nuclei, and the liver. The results from family studies further supported the diagnosis of autosomal recessive inheritance of ACP in this pedigree and molecular genetic testing was initiated.

### Molecular genetics: detection of a CP splice-site mutation

To identify the genetic cause of ACP, hepatic cDNA analysis was carried out and RT-PCR did not show the expected full length product but showed an aberrant fragment, 100 bp shorter than the expected for the PCR product spanning from exons 13 to 16 (Supplementary Fig. 1). Sequencing of the aberrant fragment showed absence of exon 14 in all biochemically affected family members. No other mutations were detected.

**Supplementary Figure 2:**
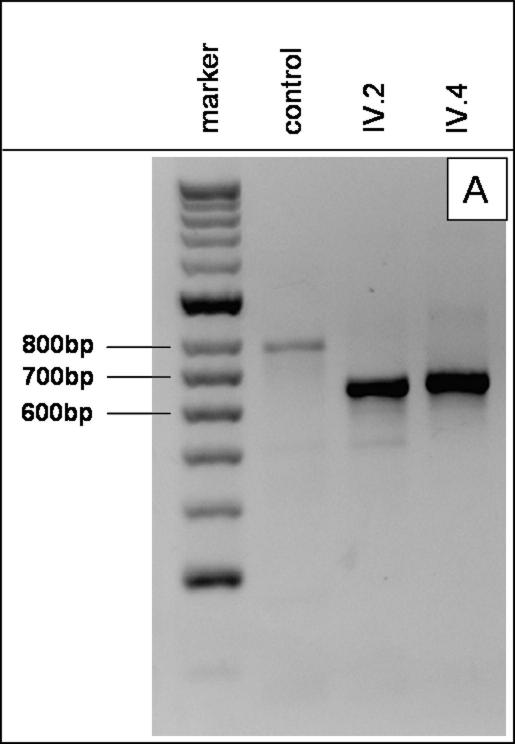
**RT-PCR results**. Skipping of exon 14 is the functional consequence of homozygosity for c.2813+1G>T as shown by cDNA amplification. Amplification of a cDNA fragment spanning Exon 13 – Exon 16 shows that the apparent size of the resulting RT-PCR product from RNA extracted from an affected individual‘s liver is approximately 100 bp shorter than expected. As a control liver cDNA from an individual with normal CP concentrations was used and showed a PCR product of the expected size (800bp). Skipping of Exon 14 was confirmed by direct sequencing of the PCR products.

Direct sequencing of genomic DNA revealed homozygosity for c.2554+1G>T in intron 14 of the CP gene in all biochemically affected family members. The mother (III.1) and one unaffected brother (IV.3) were heterozygous carriers, whereas the other brother had the wild-type sequence on both alleles. The c.2554+1G>T mutation of the CP gene was not found in 100 control chromosomes by PCR amplification, restriction fragment length polymorphism, and D-HPLC (Supplementary Fig. 2). Genetic and biochemical findings in this family show segregation of this novel mutation with ACP.

### Functional consequences

The c.2554+1G>T mutation, which was shown to cause skipping of exon 14 is predicted to preserve the open reading frame and resulted in the loss of 43 amino acids spanning residues G809–G852 with a molecular weight of 4.6 kDa. This was confirmed by SDS–PAGE and Western blotting of proteins extracted from liver biopsies of genetically affected family members and a healthy control, which showed major immunoreactive bands at the expected molecular weights (Fig. 4A).

**Supplementary Figure 3:**
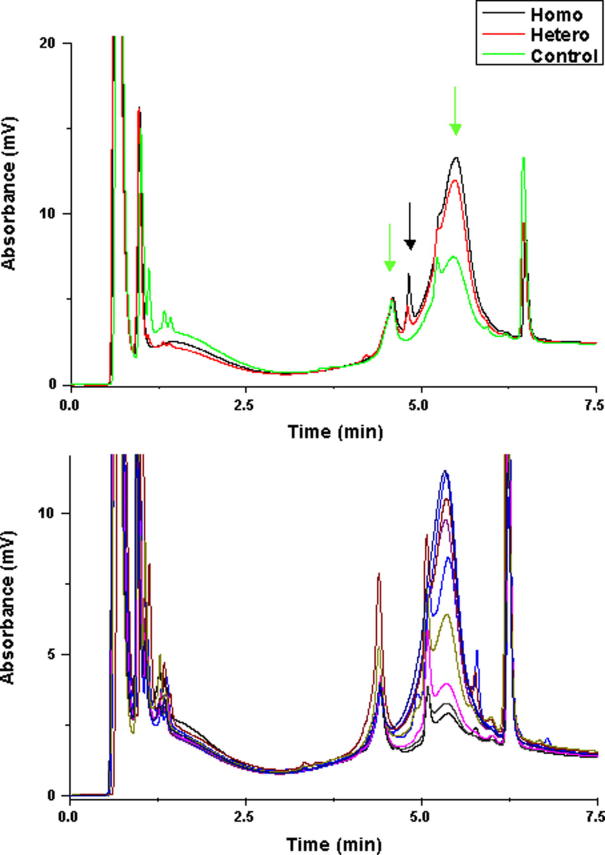
**High performance liquid chromatograms of*Rsa*I digested PCR products from individuals homozygous (black), heterozygous (red) and wild-type (green) for c.2813+1G>T of the CP gene (upper panel)**. Homozygosity for c.2813+1G>T creates an additional *Rsa*I cleavage cut site within the PCR fragment containing Exon 14, resulting in an additional fragment (black arrow) after *Rsa*I digestion. 100 control chromosomes were genotyped and no variant was detected, runs of 9 control persons are shown in the lower panel.

A structural homology model of the mutant protein that appears to be expressed in the liver of genetically affected individuals was constructed (Supplementary Fig. 3). Our model suggests that the missing region from G809–G852 is located within domain 5 (D724–P903) of CP, which contains neither a copper-binding site nor the ferroxidase site [23]. Analysis of the model suggests that rearrangement of the termini flanking the deleted residues would require a cumulative shift in their positions of approximately 20 Å. The abnormal CP, where the open reading frame and all copper-binding sites are putatively preserved, is expressed in hepatocytes, but undetectable in plasma.

### Hepcidin in ACP

To study the pathogenesis of iron overload in ACP, mRNA of the key regulator of iron metabolism hepcidin was quantified by real time PCR in liver biopsies. To determine if the observed increase in hepcidin expression was appropriate for the degree of iron overload, hepcidin/log (ferritin) ratios were calculated. As shown in Fig. 4B mean hepcidin mRNA levels and hepcidin/log (ferritin) ratios are higher in ACP, dysmetabolic siderosis, and patients with fatty liver disease as compared to patients with C282Y homozygous hemochromatosis or viral hepatitis.

### Course on iron chelation treatment

To treat iron overload associated with ACP, iron chelation therapy with deferasirox was started in all three homozygotes for the *CP* splice-site mutation with initial doses of 17–20 mg/kg bodyweight per day. Treatment duration ranged from 1 week to 5 months. In the index case deferasirox dose was reduced from 20 to 15 mg/kg/day after 4 weeks due to diarrhea and after 3 months treatment was stopped because of anemia (hemoglobin 9.9 g/dl). Patient IV.4 also developed anemia (hemoglobin 10 g/dl) and treatment was stopped after 5 months due to a gradual increase in creatinine up to 1.3 mg/dl. Treatment was discontinued after one week in patient IV.5 because of a skin rash.

**Fig. 2:**
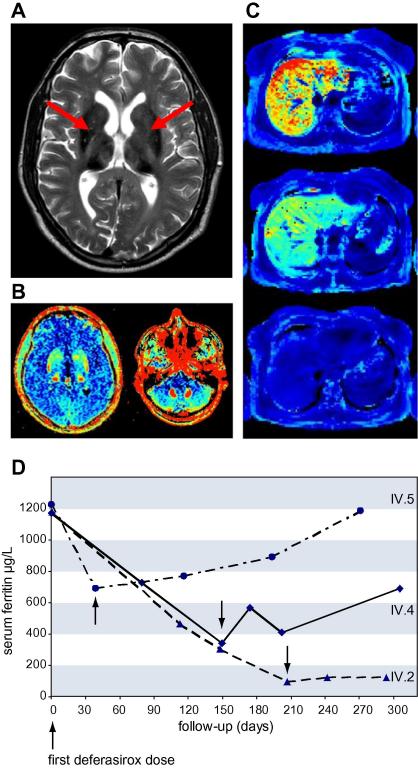
**Iron accumulation and results from deferasirox treatment.** (A) T2 brain MRI of patient IV.4 shows a marked hypodensity in the area of the basal ganglia indicating severe iron accumulation (arrows). (B) T2∗ maps of the brain confirm the significant iron accumulation in the basal ganglia and show iron loading in the dentate nucleus (red colour indicates areas with a high and dark blue colour those with a low tissue iron concentration). (C) Abdominal T2∗ maps of patient IV.4 show liver iron overload of approximately 250 μmol Fe/g liver tissue before deferasirox treatment. After 2 months of treatment a reduction in liver iron concentration was found and no visible liver iron remained after 5 months of treatment (25 μmol Fe/g liver tissue). (D) Serum ferritin concentrations rapidly decreased during deferasirox treatment. Arrows indicate the first visit after cessation of iron chelation therapy.

**Fig. 3:**
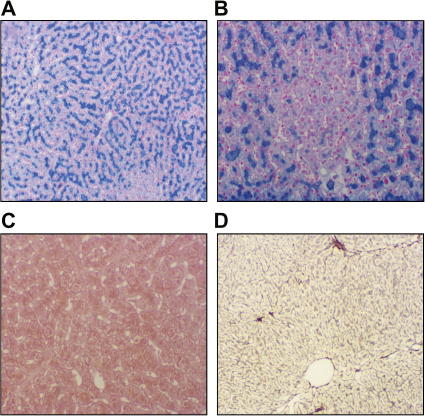
**Liver histology shows a normally structured liver with severe hepatocellular iron over-load.** (A) Perls’ stained liver biopsy of the index case with grade III iron accumulation in hepatocytes and (B) an iron free focus. (C) HE-stained liver biopsy of the index case showing normally structured liver lobes without steatosis or inflammation but with hemosiderin pigment in hepatocytes. (D) Reticulin-stained liver biopsy shows minimal fibrosis (grade I). All pictures were captured using an Olympus BH2 microscope (Olympus, Vienna, Austria) with a Jenoptik Progress C12 digital camera and Progress Capture Pro 2.5 software (Jenoptik, Jena, Germany). Panels A and D 100×, panels B and C 200×.

**Fig. 4:**
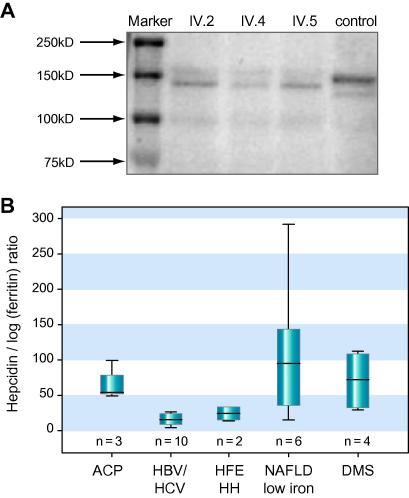
**Proteins extracted from liver biopsies of genetically affected family members.** (A) Western blotting of protein extracts from a control liver shows an immunoreactive band at 150 kDa. In protein extracts from liver biopsies of genetically affected family members the strongest signal has an apparent molecular weight of 145 kDa and is slightly lower than the major band in the control liver extract. The difference corresponds to the predicted loss of 4.6 kDa in molecular weight of the protein translated from the abnormal transcript in c.2554+1G>T homozygotes. (B) Hepcidin mRNA concentrations in liver biopsies of the three ACP patients are comparable to those in patients with non alcoholic fatty liver disease (NAFLD) or dysmetabolic siderosis (DMS) but higher than in patients with HFE hemochromatosis (HFE HH) or viral hepatitis.

Serum ferritin concentrations decreased in all patients whereas serum iron, transferrin, and transferrin saturation remained unchanged during deferasirox therapy (Fig. 2D). Non-invasive iron quantification by MRI showed a marked decrease in liver iron concentration after 3 months in the index case and in patient IV.4. After 5 months of treatment T1 and T2∗ relaxation times in patient IV.4 increased from 390 to 513 ms (normal 570 ± 40 ms) and from 1.8 to 10.9 ms (normal 24 ± 6 ms), respectively. However, there was no imaging evidence for significant effects on brain iron overload during iron chelation therapy in both patients. Accordingly, neurological symptoms remained stable during the follow up. Both patients reported a transient reduction in their insulin demands on deferasirox. Iron chelation therapy with deferasirox is effective in reducing hepatic iron concentrations and improves endogenous insulin production, but has no short term effect on brain iron accumulation.

## Discussion

CP is a multi-copper ferroxidase essential for iron homeostasis which facilitates cellular iron efflux [7,24]. Lack of functional CP is typically associated with iron overload in the liver, pancreas, and the basal ganglia as illustrated in the reported cases. Retinal degeneration has a penetrance ranging from 75% to 93% in patients with ACP and retinal pigment epithelium changes were present in patients IV.4 and IV.5 [4]. It is unclear if the incomplete penetrance of neurological symptoms and diabetes in our patients is a result of disease modifiers or reflects the natural course of ACP. Both clinically affected siblings are diabetic since their twenties, whereas the youngest genetically affected sibling is asymptomatic and not diabetic at the age of 37, which suggests the presence of modifiers that affect disease expression.

In agreement with previous reports of ACP, none of the genetically affected individuals had evidence of impaired biochemical liver function or advanced fibrosis despite the significant hepatocellular iron overload that exceeded the thresholds for hepatic fibrosis established in hemochromatosis cohorts [13,25,26]. This observation suggests that iron is only a cofactor in hepatic fibrogenesis [27].

Liver histology showed the presence of iron in hepatocytes, whereas no iron was found in macrophages. Moreover, magnetic resonance imaging also showed the absence of iron overload in the spleen, suggesting that iron overload in Kupffer cell is a late event in ACP [15]. This observation suggests that CP is important for hepatocellular iron efflux, whereas CP-independent mechanisms for iron export from macrophages exist. If CP was indispensable for iron export from recycling macrophages, one would expect more pronounced anemia and even higher sTfR concentrations.

In our ACP patients, stainable iron was restricted to hepatocytes, in comparison to patients with advanced hemochromatosis or transfusional iron overload where the iron storage typically affects parenchymal cells and macrophages. The decline of serum ferritin on deferasirox treatment in ACP patients was faster than in patients with ß-thalassemia, which suggests that iron-loaded hepatocytes are the main source of serum ferritin and hepatocellular iron is more accessible to deferasirox than macrophageal iron. The prompt increase in serum ferritin after cessation of deferasirox treatment may indicate a redistribution of iron, since the re-accumulation of food iron in such a short time appears unlikely.

Hepatocellular iron overload induces expression of the peptide hormone hepcidin in normal individuals, which causes ferroportin degradation. This results in cellular iron retention and reduced iron absorption [28,29]. The observation that hepatic hepcidin mRNA expression in ACP patients and in dysmetabolic siderosis patients are comparable, but low in HFE hereditary hemochromatosis, suggests that CP but not HFE is dispensable for iron-mediated hepcidin regulation [30]. We found higher hepcidin mRNA expression in ACP than in viral hepatitis patients where hepcidin expression is suppressed [31], which also suggests that hepcidin responds normally to iron in ACP. Normal hepcidin regulation in ACP supports the concept that regional iron accumulation in affected organs results from disturbed cellular iron metabolism rather than from alterations in total body iron homeostasis.

The functional consequence of the splice-site mutation c.2554+1G>T is the skipping of exon 14, which preserves the open reading frame. The structural model suggests that the abnormal protein folds into a potentially functional protein, but such a significant structural change within domain 5 of CP is likely to affect the stability of the variant protein, resulting in intracellular retention. The phenotypic similarity with CP nonsense mutations further suggests that this variant is dysfunctional [32].

Skipping of exon 14 is also predicted to affect the alternative transcript in the brain, where activation of a cryptic splice-site results in direct splicing of exons 18–20 [6]. Iron accumulation in the basal ganglia of c.2554+1G>T homozygotes suggests the absence of functional GPI-anchored CP and supports the importance of correct protein folding for the activity and stability of GPI-anchored CP. Ferroxidase activity is required to stabilize the iron export pump ferroportin in cells expressing GPI-anchored CP, which provides a molecular explanation for cellular iron retention in ACP [9].

Iron-mediated lipid peroxidation and oxidative stress are the main causes of neurodegeneration secondary to brain iron accumulation [33,34]. To reduce iron toxicity chelation therapy was introduced in ACP. The iron chelator deferoxamine was effective in reducing hepatic iron overload and leading to a partial improvement of neurological symptoms and brain iron accumulation, as reported in a single case report [16]. The positive effect of deferoxamine on central nervous symptoms and signs was not found in subsequent studies [14,15], which highlights the low permeability of the blood–brain barrier for deferoxamine. Despite the lower molecular weight and more lipophilic properties of deferiprone, this orally bioavailable iron chelator had no beneficial effect in ACP in a previous report [14]. In agreement, we found no improvement of neurological symptoms or brain iron accumulation during deferasirox treatment over a period of several months. Deferasirox could still be an effective brain iron chelator, whose clinical benefit might be missed after a short term treatment. By comparison, the neurological improvement on zinc-sulphate was found after 15 months of treatment in a patient with ACP [35]. In contrast, hepatic iron concentration and insulin requirements improved in both patients who were treated for 3 and 5 months, respectively. Taken together, short term deferasirox is effective in reducing hepatic iron overload, but ineffective for the treatment of brain iron accumulation. In all patients, side effects prohibited the long term therapy that may be required to mobilize iron from the brain. Given the high efficacy of a high dose of deferasirox, a low dose of the iron chelator may have a more favourable side effect profile, which in turn may allow for a long term and combination therapy. Recent studies in an animal model of Friedreich’s ataxia suggest that the combined use of pyridoxal isonicotinoyl hydrazone and deferoxamine is an effective treatment of iron accumulation in glial cells [36]. Further work is required to determine if similar strategies can be developed for patients with ACP and other neurodegenerative diseases that are associated with brain iron accumulation.

## Financial disclosure

This work was supported by the Austrian Science Fund Project 19579 to H.Z. The authors report no commercial relationships that might pose a conflict of interest in connection with this manuscript.

## Figures and Tables

**Supplementary Figure 4:**
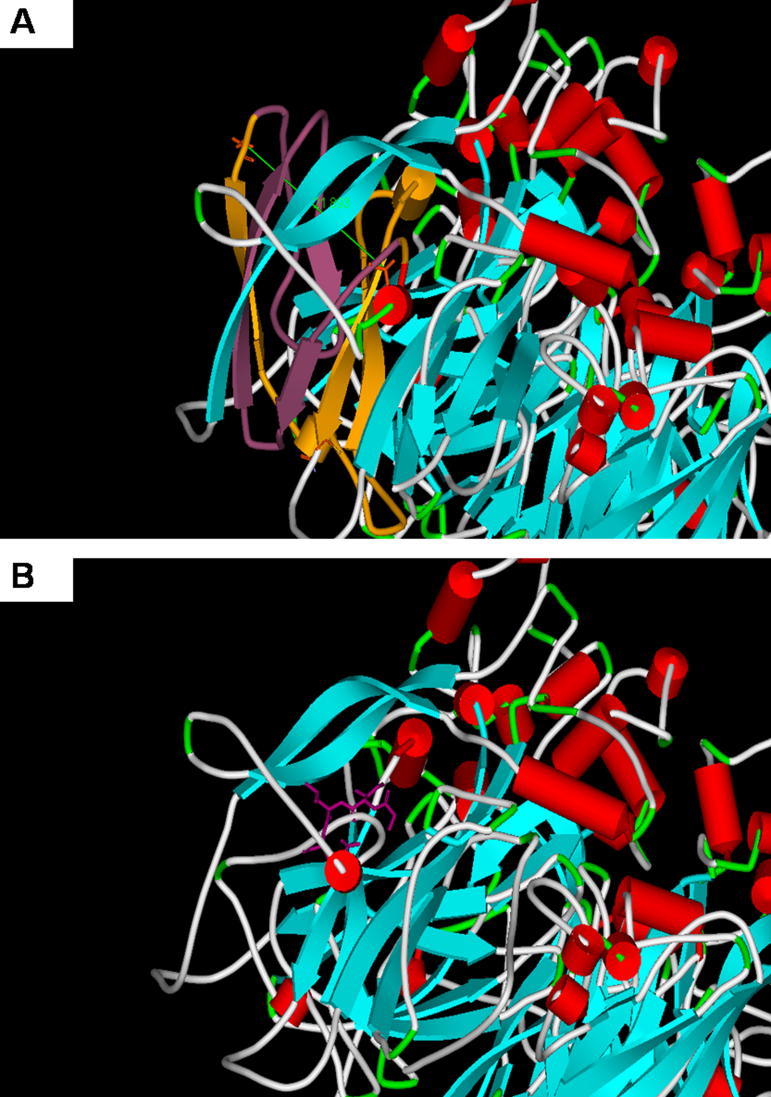
**Homology model of CP variant.** (A) View based on the published structure of human CP (PDB 2J5W:A). Deletion of residues 809–852 of CP (purple), would create a gap of ∼21.6 Angstrom between the Cα at the ends of the chains (flanking residues are coloured orange, to guide the eye). (B) A homology model of variant CP based on the published structure of human CP. In the truncated variant a local rearrangement of the flexible protein structures neighbouring the missing domain is required to form the putative-folded species. Side-chains adjacent to residues 809 and 842 in wild type CP (the residues coded by exon 14 are missing in the ACP variant) are shown in purple, to help locate the variant sequence that is formed by the deletion and to highlight the potential structural rearrangement.

**Table 1:** **Demographic, hematological, serum iron parameters, hepatic, and genetic data of members of the affected family at the time of first presentation**.

*Abbreviations:* f, female; m, male; Hb, hemoglobin; TfS, transferrin saturation; SC, serum ceruloplasmin; HC, hepatic copper concentration; HIC, hepatic iron concentration; n.d., not determined.
