# Supplementary material for: Hepatic but not brain iron is rapidly chelated by deferasirox in aceruloplasminemia due to a novel gene mutation
Source: J Hepatol. 2010 Dec;53(6):1101–7. doi: 10.1016/j.jhep.2010.04.039 (PMC2987498; doi:10.1016/j.jhep.2010.04.039)
Supplement: Supplementary data 1 [file mmc1.doc]

**Supplementary material**

Figure S1. RT-PCR results. Skipping of exon 14 is the functional consequence of homozygosity for c.2813+1G>T as shown by cDNA amplification. Amplification of a cDNA fragment spanning Exon 13 – Exon 16 shows that the apparent size of the resulting RT-PCR product from RNA extracted from an affected individual‘s liver is approximately 100 bp shorter than expected. As a control liver cDNA from an individual with normal CP concentrations was used and showed a PCR product of the expected size (800bp). Skipping of Exon 14 was confirmed by direct sequencing of the PCR products.

Figure S2. High performance liquid chromatograms of *Rsa*I digested PCR products from individuals homozygous (black), heterozygous (red) and wild-type (green) for c.2813+1G>T of the CP gene (upper panel). Homozygosity for c.2813+1G>T creates an additional *Rsa*I cleavage cut site within the PCR fragment containing Exon 14, resulting in an additional fragment (black arrow) after *Rsa*I digestion. 100 control chromosomes were genotyped and no variant was detected, runs of 9 control persons are shown in the lower panel.

Figure S3. Homology model of CP variant. (A) View based on the published structure of human CP (PDB 2J5W:A). Deletion of residues 809-852 of CP (purple), would create a gap of ~21.6 Angstrom between the Cα at the ends of the chains (flanking residues are coloured orange, to guide the eye). (B) A homology model of variant CP based on the published structure of human CP. In the truncated variant a local rearrangement of the flexible protein structures neighbouring the missing domain is required to form the putative-folded species. Side-chains adjacent to residues 809 and 842 in wild type CP (the residues coded by exon 14 are missing in the ACP variant) are shown in purple, to help locate the variant sequence that is formed by the deletion and to highlight the potential structural rearrangement.

Table S1. Primers and PCR conditions for RT-PCR

| Primer | forward | Reverse | PCR product size | | annealing temaprature | |
| --- | --- | --- | --- | --- | --- | --- |
| RT-PCR 1 | TCATTTCTTCTCAGGCTCCAA | TCAGCACACATGGAGAGTCC | 589 | bp | 65 | °C |
| RT-PCR 2 | GGTCTGGCTTGGGTTTTTAG | GAGCATCAATGTGGGAATGG | 573 | bp | 64 | °C |
| RT-PCR 3 | CCGAGAATTTGTGGTGATGTT | TTAGTGGGTTCCACAGCAGA | 574 | bp | 63 | °C |
| RT-PCR 4 | CCGTGGGAAGCATGTTAGAC | TGAGTCACTTCCAGGTGCTG | 543 | bp | 65 | °C |
| RT-PCR 5 | GAATGGACTGTCCCCAAAGA | CCCATGGGCATGTATTGAGT | 533 | bp | 63 | °C |
| RT-PCR 6 | GGAGAACGGAGAGACACAGC | AAAGTCCCCTCTGTGTCAGG | 510 | bp | 63 | °C |
| RT-PCR 7 | TCATGCAGATGTTGGAGACAA | CCTAGTGTACAAGTGTCAGTCATGT | 573 | bp | 64 | °C |
| RT-PCR 8 | ATGCACGTGGGAGATGAAG | AAGCTATGGCCGTGAAAATG | 502 | bp | 62 | °C |

Table S2. Primers and PCR conditions for genomic DNA sequencing

| Primer | forward | reverse | PCR products size |
| --- | --- | --- | --- |
| PCR 11 | GGTCCTGGAAAGTCTGTGAG | CACCCAACACATTCTGCTAC | 372 bp |
| PCR 12 | GCCATGATAAGGACTTCAGC | GGACAAGTTCCTAGGAAGTG | 463 bp |
| PCR 13 | CCTACTTGGATAGAAGAGTG | CTGCAGGTAGCATCACATC | 340 bp |
| PCR 14 | CATGGGGGAGAGAAACAAG | GCCTGTTAAAATGCACCACC | 267 bp |
| PCR 15 | CCTAGTTATCAAGGCTGTGTG | CGTAGAATTGGACCACAGG | 305 bp |
| PCR 16 | GGTGCAAAGTCTCAGTTCTC | CAAAGTGAGGCAGAAGTGG | 410 bp |
| PCR 17 | TTCTTTGAAATAGCTATTGG | CAATCCATGATTGAATCCTG | 366 bp |
